# The prognostic value of a normal oral glucose tolerance test in pregnant women who tested positive at screening: a validation study

**DOI:** 10.1186/1758-5996-4-10

**Published:** 2012-04-03

**Authors:** Patricia M Rehder, Belmiro G Pereira, João Luiz Pinto e Silva

**Affiliations:** 1 School of Medical Sciences, University of Campinas (UNICAMP), Campinas, SP, Brazil; 2 Department of Obstetrics & Gynecology, School of Medical Sciences, University of Campinas, Campinas, São Paulo, Brazil; 3 The Prof. Dr. José A. Pinotti Women's Hospital, a Teaching Hospital of the University of Campinas (UNICAMP), Campinas, São Paulo, Brazil

**Keywords:** Gestational diabetes mellitus, Risk factors for gestational diabetes mellitus, Perinatal, Outcomes in gestational diabetes mellitus, Prenatal care

## Abstract

**Background:**

Controversies surround a diagnosis of gestational diabetes mellitus (GDM). The objective of this study was to evaluate the oral glucose tolerance test (OGTT) for the prediction of adverse gestational and perinatal outcomes in pregnant women with a positive screening test for diabetes mellitus and a negative diagnosis, i.e. a normal 3-hour OGTT.

**Methods:**

This validation study evaluated 409 pregnant women who tested positive for diabetes mellitus at screening. Perinatal and maternal outcomes were considered. Sensitivity and specificity were calculated for each of the values of the OGTT as a diagnostic test, with the gold standard being perinatal outcome.

**Results:**

The most frequent risk factors were obesity, arterial hypertension and advanced maternal age. The most common neonatal outcomes were large-for-gestational-age infants, Cesarean delivery and preterm birth. A fasting blood glucose level of 87 mg/dL was the most powerful predictor of adverse perinatal outcome.

**Conclusions:**

At the cut-off level adopted by the American Diabetes Association, gestational OGTT was able to successfully identify in which pregnant women outcome would be unfavorable.

## Background

Many controversies surround the diagnosis of gestational diabetes mellitus (GDM). In 1998, the American Diabetes Association (ADA) recommended the adoption of an oral glucose tolerance test (OGTT) using 100 or 75 g of dextrosol with well-defined cut-off limits for glucose levels at fasting and following a glucose overload: fasting < 95 mg/dL; 1 h < 180 mg/dL; 2 h < 155 mg/dL; and 3 h < 140 mg/dL [1]. GDM is diagnosed when two or more values are found to be above the established cut-off limits. These recommendations were based on studies conducted by O'Sullivan and Mahan, published in 1964 [2] and adapted by Carpenter and Coustan in 1982 [3]. They have been maintained for the past 10 years [4].

The International Association of Diabetes and Pregnancy Study Groups (IADPSG) recommended the adoption of certain markers for screening. If one of these markers is present, an oral glucose tolerance test is then performed. According to the guidelines proposed by IADPSG, only one value above the cut-off limit in the 3-hour OGTT is sufficient to justify a diagnosis of GDM. If applied, this criterion will lead to a diagnosis of GDM in 18-20% of the entire obstetric population [5].

In parallel, a diabetes study group in Brazil developed a consensus statement on the diagnosis and treatment of diabetes in pregnancy. This consensus established the 2-hour OGTT with 75 g of dextrosol as the standard diagnostic test, with a diagnosis of GDM being established when at least two values are above the cut-off limits, which coincide with those of the ADA proposal [4-6].

The thresholds adopted by the ADA for the 3-hour OGTT are useful for diagnostic purposes in populations similar to that of Brazil in which the prevalence of GDM is moderate when compared to other, previously tested populations such as those of certain European countries and the United States. The results established in those populations formed the basis for defining the current cut-off limits [4].

Screening, however, is still carried out based on the presence of risk factors and by measuring fasting blood glucose levels. If a woman has any of the risk factors or if her fasting glucose level is ≥ 85 mg/dL, she is considered to have tested positive for GDM at screening [6].

The present study was performed to evaluate the prevalence of maternal and neonatal complications in women who screened positive for GDM but who had a normal 3-hour OGTT. An additional objective was to assess the accuracy of the OGTT for the prediction of adverse perinatal outcomes in this same population.

## Methods

A study was conducted to validate a diagnostic test (3-hour OGTT) in pregnant women who had screened positive for GDM but who had a normal 3-hour, 100-gram OGTT. The study was carried out at the *Prof Dr José A. Pinotti *Women's Hospital, a teaching hospital at the State University of Campinas (UNICAMP), between January 2000 and December 2009.

Sample size was calculated at 400 tests from 400 pregnant women. Overall, 409 tests from 409 patients were evaluated. The inclusion criteria consisted of a positive screening test for diabetes mellitus and an OGTT with either only one value above the cut-off level or with all values within the normal range (e.g. fasting glucose 95 mg/dL, 1-hour 180 mg/dL, 2-hour 155 mg/dL, 3-hour 140 mg/dL). To be considered positive at screening, there had to be at least one risk factor (age ≥ 35 years, previous GDM, family history of DM, previous macrosomic newborn infant, BMI ≥ 25 and chronic hypertension) or fasting blood glucose had to be ≥ 90 mg/dL.

**Figure 1:**
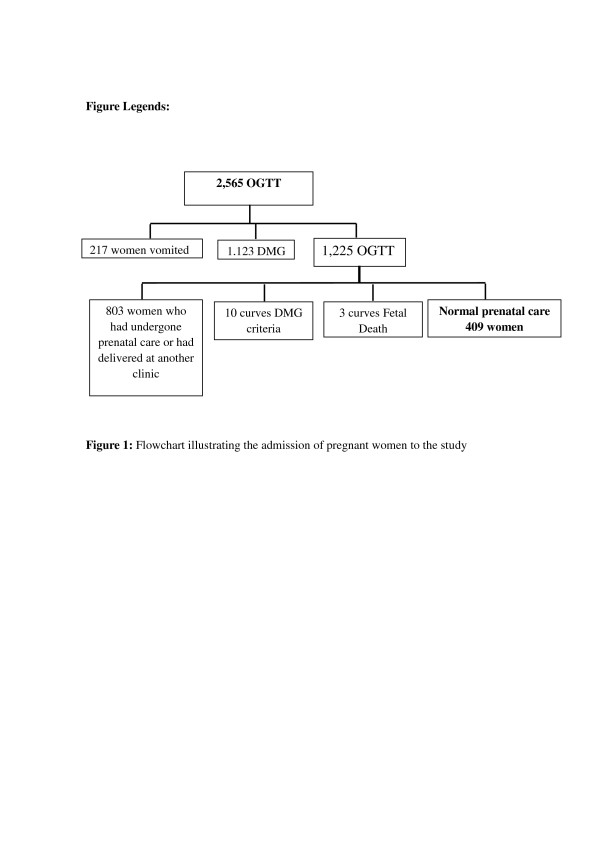
**Flowchart illustrating the admission of pregnant women to the study**.

Overall, 2,565 OGTT were performed during the study period; however, 217 of the women vomited during the test and it had to be stopped. In these cases, no further gestational OGTT was performed. Of the remaining 2,348 tests, 1,123 women received a diagnosis of gestational diabetes mellitus. With respect to the other 1,225 OGTT performed, 803 referred to women who had undergone prenatal care or had delivered their infant at another clinic. Of the remaining 422 tests, criteria for diagnosis were present in 10 cases, while 412 were considered normal. Of these, three were later excluded from the data analysis because of fetal death. Therefore, 409 curves were included in the study (Figure 1).

The maternal variables evaluated were: age, body mass index (BMI), weight, height, number of pregnancies, parity, previous Cesarean delivery and gestational age at time of delivery. The perinatal outcomes analyzed were: macrosomia (birthweight > 4,000 grams), large-for-gestational-age infants (LGA - birthweight above the 90^th ^percentile for GA at delivery), hydramnios (amniotic fluid index above the 90^th ^percentile for GA), neonatal hypoglycemia (newborns with capillary blood glucose < 40 mg/dL), neonatal hyperbilirubinemia (venous bilirubin levels > 16 mg/dL), 5-minute Apgar score < 7, neonatal respiratory distress syndrome and C-section.

First, a descriptive analysis was performed and prevalence rates of maternal and perinatal variables were calculated, together with their 95% confidence intervals (95% CI). Receiver operating characteristic curves (ROC) were constructed for the OGTT as a diagnostic test, with the gold standard being the perinatal variables. The area under the ROC curve was considered statistically significant when it differed by 50%. The highest sensitivity and specificity values were considered the optimal cut-off limits for the prediction of adverse perinatal outcomes.

Data were stored and analyzed using the Excel software program, version 6.0 b (Microsoft Corporation), while the consistency of data was verified by performing visual and statistical analyses in duplicate. The Internal Review Board of this Medical School approved the study under approval letter # 1131/2008.

## Results

The median age of the women in this study was 29 years (range 15 to 45 years). Mean maternal weight was 74 kg (range 46.5 to 143.3 kg), while mean BMI was 29. Median gestational age at birth was 38-39 weeks. Overall, 306 pregnant women (74.8%) had had two or more previous pregnancies, while 98.7% had had at least one previous delivery and 64% had had a previous C-section.

The most common risk factor was overweight, defined as BMI ≥ 25 kg/m^2^, with 60% of the women falling into this category. Another important risk factor was chronic hypertension (23%), followed by maternal age (20%) and a family history of diabetes (20%). Thirty-seven percent of the women had only one risk factor, while around one-third had two or more risk factors. Fasting blood glucose level was abnormal in about 10-12% of the women; however, this constituted the only risk factor in less than 2% of patients. Seventy-nine patients had no risk factor and their fasting glucose levels were normal, with only signs suggestive of overt diabetes such as fetal macrosomia, excessive weight gain or hydramnios being present (Table 1).

**Table 1:** Prevalence of risk factors and fasting glucose ≥ 90 mg/dL (n = 409)

Risk factor	N	%
Fasting Glucose ≥ 90 mg%	47	11,5
Age ≥ 35 years	81	19,8
Previous GDM	13	3,2
Family history of DM	81	19,8
Previous macrosomia newborn	30	7,3
Body mass index (BMI) ≥ 25	244	59,8
Chronic hypertension	94	23,0
Suggestive clinical signs	79	19,3

*GDM *gestational diabetes mellitus

*DM *diabetes mellitus

There was a high prevalence of LGA infants (19.3% overall), an outcome that occurred in one-third of the women with an abnormal fasting glucose level and in about one-fifth of those with only one risk factor. LGA infants were also born to around a quarter of women with two or more risk factors. No correlation was found between any of the other outcomes and the presence or absence of risk factors or serum glucose screening values (Table 2).

**Table 2:** Distribution of perinatal outcomes according to risk factors (RF) and fasting glucose (FG)

			Outcomes			
**Risk Factor**	**Macrosoa** **n/%**	**LGA** **n/%**	**Hydramnio** **n/%**	**Hypoglycemia** **n/%**	**RDS** **n/%**	**Hyperbilirubinemia** **n/%**
						
**Fasting Glucose**						

**2 or more **(132)	16/12.1	34/25.7	11/8.3	2/1.5	3/2.2	3/2.2
**Only one **(151)	13/8.6	32/21.2	9/5.9	6/3.9	7/4.6	5/3.3
**Glucose and others **(41)	3/7.3	6/14.6	1/2.4	0/0	2/4.8	1/2.4
**Only Glucose **(6)	0/0	2/33.3	1/16.6	0/0	0/0	0/0
**None **(79)	3/3.7	5/6.3	4/5.0	4/5.0	3/3.7	0/0

**Total (409)**	35/8.5	79/19.3	26/6.3	12/2.9	15/3.6	9/2.2

*RF *risk factors

*LGA *large for gestational age

*RDS *respiratory distress syndrome

**Figure 2:**
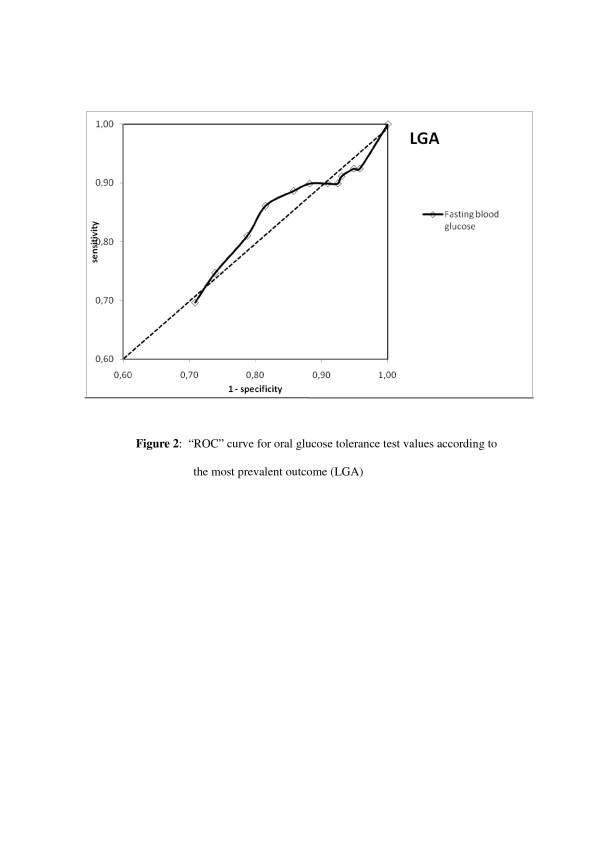
**"ROC" curve for oral glucose tolerance test values according to the most prevalent outcome (LGA)**.

A ROC curve was constructed for fasting blood glucose levels. As shown in Table 3, sensitivity and specificity for the fasting blood glucose curve were highest at the value of 87 mg/dL for the prediction of LGA, which was the most prevalent neonatal variable. However, the area under the curve does not justify changing the current cut-off points in the OGTT to this value (Figure 2).

**Table 3:** Sensitivity and specificity of OGTT in predicting large for gestational age newborns

Fasting glucose	Sensitivity (95% CI)	Specificity (95% CI)
≤ 84 mg/dl	69.6 (59.5-79.8)	29.1 (24.2-34.0)
85 mg/dl	74.7 (65.1-84.3)	26.1 (21.3-30.8)
86 mg/dl	81.0 (72.4-89.7)	21.2 (16.8-25.6)
**87 mg/dl**	**86.1 (78.4-93.7)**	**18.5 (14.3-22.7)**
88 mg/dl	88.6 (81.6-95.6)	14.2 (10.5-18.0)
89 mg/dl	89.9 (83.2-96.5)	11.8 (8.3-15.3)
90 mg/dl	89.9 (83.2-96.5)	9.1 (6.0-12.2)
91 mg/dl	89.9 (83.2-96.5)	7.6 (4.7-10.4)
92 mg/dl	91.1 (84.9-97.4)	7.0 (4.2-9.7)
93 mg/dl	92.4 (86.6-98.2)	5.2 (2.8-7.5)
94 mg/dl	92.4 (86.6-98.2)	4.2 (2.1-6.4)
≥ 95 mg/dl	100.0	0.0

*95% CI *95% confidence interval

*OGTT *oral glucose tolerance test

With respect to the remaining OGTT values (60', 120', 180'), none of the values of the area under the ROC curve differed by more than 50%; therefore, it was concluded that there was no correlation with the perinatal outcomes evaluated.

## Discussion

In this study population, the most prevalent risk factors were obesity or overweight, maternal age > 35 years, chronic hypertension and excessive weight gain during pregnancy. These data are in agreement with reports published in the medical literature over the years [7,8].

Abnormal fasting blood glucose levels were not a relevant indication for performing an OGTT. This finding contradicts what has been established in the literature in which this is highlighted as constituting a basic parameter [9]. However, when blood glucose is associated with a risk factor, it gains importance as an indication for performing an OGTT [10,11]. It should be emphasized that clinical signs during pregnancy such as maternal weight gain, fetal weight and increased amniotic fluid are strongly related to glucose intolerance and should signal a need to request an OGTT. In the population studied, 32.3% of the sample fulfilled these criteria. This finding is supported in the literature by studies such as those carried out by Ferrara and Agarwal [12,13].

When the results of the OGTT were compared with the outcome of pregnancy as the gold standard, it was found that there are no cut-off limits other than those already established that are capable of forecasting significant differences, except for fasting blood glucose levels for which the best cut-off limit would be 87 mg/dl.

It must be noted that from a methodological point of view, there were few useful values available with which to construct the ROC curve, which may have hampered analysis to a certain extent. To construct these ROC curves, cumulative numbers were used that focused on one extreme, generally the lower limits, and with this technique the cut- off limit is shifted to lower numbers.

Another limitation of the study was the fact that neonatal variables were used as the gold standards, and these are likely to be affected by several other factors, unlike the markers that have been used specifically for GDM and dysglycemia such as cord blood C-peptide and the percentage of body fat in the neonate [14]. These markers are highly specific for GDM and metabolic disturbances, and are used to identify pregnant women in whom metabolic changes are minimal [15]. However, in terms of perinatal care in developing countries where resources are sparse, this strategy of placing the emphasis on clinical data appears extremely timely and useful.

Naturally, in studies involving larger samples such as the HAPO study, values other than those obtained in the present study may be found. In our study, there was a high prevalence of LGA infants born to women with a normal OGTT (according to the ADA guidelines) and risk factors such as advanced maternal age, obesity and chronic hypertension, associated or not with abnormal fasting blood glucose [4,11,16].

## Conclusion

In conclusion, 3-hour OGTT, as recommended by the ADA [4] and adopted for use in Brazil, is adequate for establishing a diagnosis of GDM in this population and there is no need to change the current established cut-off limits.

## Competing interests

The authors declare that they have no competing interests.

## Authors' contributions

All the authors participated equally in the development of this study. All authors also read and approved the final manuscript.
